# The overshoot phenomenon in step‐and‐shoot IMRT delivery

**DOI:** 10.1120/jacmp.v2i3.2607

**Published:** 2001-09-01

**Authors:** Gary A. Ezzell, Suzanne Chungbin

**Affiliations:** ^1^ Department of Radiation Oncology Mayo Clinic Scottsdale Scottsdale Arizona 85259

**Keywords:** IMRT delivery, quality assurance

## Abstract

The control loop in the Varian DMLC system (V4.8) requires ~65 msec to monitor and halt the irradiation of a segment, causing an “overshoot” effect: the segment ends on a fractional monitor unit larger than that planned. As a result, the actual MU delivered may differ from that planned. In general, for step‐and‐shoot treatments, the first segment receives more, the last receives less, and intermediate segments vary. The overshoot for each segment (ΔMU) is small, approximately 0.6 MU at 600 MU/min Our IMRT planning system (Corvus) produces plans often having more than 20% of the segments with less than 1 MU/segment. Such segments may be skipped if the ΔMU exceeds the segments’ planned MU. Furthermore, QA filming often requires reducing the total MU by a factor of 4–6, increasing the potential for dosimetric error. This study measured ΔMU over a range of MU/min and MU/segment. At >5 MU/segment, the ΔMU was stable, corresponding to a delay of 62 msec. ΔMU became larger and more variable at <1 MU/segment. The behavior was modeled in a computer program that predicted the change in delivered MU/segment and total change in delivered MU to each beamlet. Beams were analyzed for patients receiving 5 field prostate or 9 field head and neck treatments. At 400 MU/min, 28% and 16%, respectively, of the planned segments were skipped. For QA filming, up to 75% of the segments were skipped. The cumulative error averaged <0.1 MU/beamlet, but individual beamlets had errors exceeding 200%. The effect is most significant for low dose regions. Recommendations are given for deciding when to treat or do QA studies with lower MU/min. In general, treatments are not significantly affected, but QA films taken at reduced MU may be improved if irradiated at lowered MU/min.

PACS number(s): 87.53.–j, 87.90.+y

## INTRODUCTION

Intensity‐modulated radiation therapy (IMRT) at our facility is currently planned with Corvus (Nomos Corporation, Sewickley, PA) and delivered on Varian accelerators (Palo Alto, CA) using dynamic multileaf collimation (DMLC). Several observations about this system led us to question if there were situations in which irradiations should be done at less than the maximum delivery rate (MU/min) available.

The first observations relate to the DMLC system. We deliver treatments in step‐and‐shoot mode, which means that the system alternates between delivering radiation with a static MLC pattern and moving to the next pattern without irradiating. The DMLC files that program the treatment index of the MLC leaf position to a fractional monitor unit (fMU), which begins at 0.00 and ends at 1.00. One can, therefore, use the same file with different total MU. The MLC workstation displays the current leaf position and accumulated fMU. The first observation is that the displayed fMU is larger than that programmed, except for the final value which is always 1.00.

**Figure 1:**
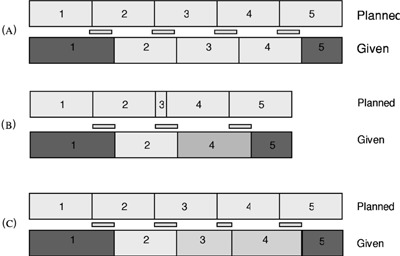
Schematic showing the effect of an overshoot in the MU delivery. (A) Overshoot is constant. Segment 1 is long, 5 is short, 2–4 are unaffected. (B) Overshoot exceeds the planned MU for segment 3, so it is skipped. (C) Overshoot is variable, so segment 3 is shorter than planned and segment 4 is longer.

This fact has been noted before,^1^ ^,^ ^2^ and can be explained as follows: the MLC controller monitors the cumulated MU, calculates the fMU, and signals the linac when to stop irradiating. This process requires 50–80 msec, and the linac delivers radiation in that interval. Thus, there is an “overshoot,” or ΔfMU, where there is more irradiation than intended. The total MU delivered is always correct because the MU1 signal terminates the beam independent of the MLC control loop. In consequence, the first segment's delivered incremental fMU is always larger than the planned, and the last is smaller. If the ΔfMU were constant, the intermediate segments would receive the correct incremental fMU, since each would begin and end with the same overshoot. In this paper, the error in delivered MU is called *δ*MU, and in this example δMU for the first segment is positive, the last is negative, and the intermediate are zero. Two facts complicate this simple picture, however. One is that the ΔfMU is not constant, and so there can be delivery errors in the intermediate segments as well as the first and last. Secondly, segments can be skipped entirely if the overshoot ΔfMU is larger than their programmed fMU. These points are illustrated schematically in Fig. 1.

**Figure 2:**
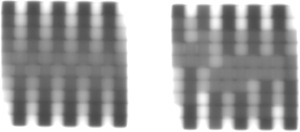
Two images taken on Kodak TL film at 15 MU of an “Up‐Down” ramp pattern. The image on the left was irradiated at 80 MU/min. The one on the right was irradiated at 400 MU/min and shows disruptions in the expected pattern.

The magnitude of the overshoot depends on the delivery rate (MU/min). In terms of absolute MU, the overshoot is simply the delivery rate times the control loop delay time. If one assumes an average delay of 65 msec, then the ΔMU is 0.65 MU at 600 MU/min, 0.43 MU at 400 MU/min etc. Figure 2 uses a pattern of alternating up‐down ramps to illustrate that the desired pattern can be disrupted if the MU/min is so fast that the ΔfMU exceeds the programmed fMU for some of the segments. These differences are small in absolute terms, however, and so one might expect that the net effects would be negligible in clinical situations.

The second observation relates to the planning system. Corvus produces plans for Varian DMLC delivery that include many segments calling for less than one MU/segment. An analysis of five field prostate plans for six patients showed that 28% of the segments had less than one MU. Similarly, plans for four patients having nine field treatments to the head and neck had 22% of the segments with less than one MU. In fact, for these prostate and head and neck patients, 28% and 16%, respectively, of the segments had less than 0.5 MU/segment. One might wonder if any delivery errors for these segments would tend to cancel or accumulate and become clinically significant.^7^ The commissioning and quality assurance (QA) studies that have been carried out here and elsewhere suggest that they do cancel, at least in the high dose, low gradient regions, for which routine ion chamber measurements are made. There remains the possibility that there may be effects in lower dose regions that have clinical importance, especially where IMRT is being used to minimize dose to nearby critical structures.

A final observation is that commissioning and QA studies are often carried out using film dosimetry with Kodak RP/V film in conjunction with film digitizers. The dynamic range of such systems is limited. One needs to reduce the MU so that the target dose is limited to about 50 cGy when using a 16‐bit digitizer, and to about 30 cGy for a 12‐bit digitizer.^3^ If the patient target dose is 200 cGy, these call for MU reduction factors of 0.25 and 0.15, respectively. For our five field prostate patients, 56% and 90% of the segments, respectively, would then have less than one MU/segment. One might wonder if apparent errors would appear as an artifact of the overshoot phenomenon when the MU are reduced for filming studies.

There are other issues with respect to small MU/segment that might affect actual dose delivery. These include the stability of the linac dosimetry system and the planning system's dose calculations and leaf sequencing.^4^ ^–^ ^6^ This study focussed only on the effect of the overshoot inherent in the linac‐MLC control loop.

The purpose of this study was to measure the overshoot (ΔfMU) as a function of delivery rate and MU/segment, and use that data to assess the error in delivered MU accumulated in individual beamlets (δMU). Ultimately, the aim was to determine if there are situations, either for patient treatment or QA studies, that require irradiation at less than maximum delivery rates in order to keep the errors negligible.

## MATERIALS AND METHODS

The overshoot was measured on a Varian 2100C accelerator using control software version 5.4 and equipped with a 52 leaf MLC, with DMLC control software version 4.8. The overshoot was measured using manually written DMLC files that moved a 1 cm leaf gap, alternately ±6 cm from the central axis. The observer recorded the fMU reported on the MLC workstation screen after each “shoot” segment, while the leaves were moving during the subsequent “step” segment. In one file, the planned fMU increased evenly: 0.1,0.2,0.3, …, 1.0; so ten segments each received a fMU of 0.10. In another, they incremented in alternating short and long segments: 0.01, 0.20, 0.21, 0.40, …, 1.0; so five segments received 0.01 and five received 0.19. In a third, the long and short segments were reversed: 0.19,0.20,0.39,0.40, …, 1.0; the increments in fMU were again 0.19 and 0.01, but the sequence was inverted. By running the files at different total MU, different MU/segments were generated and replicated several times over a range from 0.13 to 38. The tests were run at three delivery rates: 80, 240, and 400 MU/min, the last being the fastest available on our accelerators. The ΔfMU were obtained from the recorded fMU by subtracting the planned fMU (Eq. 1). The files were run a total of 47 times, leading to 139, 84, and 163 data points at 80, 240, and 400 MU/min, respectively. The results were converted to ΔMU by multiplying by the total MU used (Eq. 2).

(1) $$\Delta \text{fMU}={\text{fMU}}_{\text{delivered}}-{\text{fMU}}_{\text{planned}}$$

(2) $$\Delta \text{MU}=\Delta \text{fMU}*{\text{MU}}_{\text{total}}$$

The data were plotted for each delivery rate, and for each rate the average ΔMU was established for three MU/segment ranges: less than 1.0, greater or equal to 1, but less than 5, and greater or equal to 5.

A computer program was then written to simulate the delivery of DMLC files and compute the MU planned for each beamlet that actually delivered. It is the error in delivered MU (δMU) that is clinically interesting. The program read the DMLC file for a beam, computed the planned fMU for each segment, and added the appropriate ΔfMU, based on the planned MU/segment and the specified delivery rate. It converted the fMU to MU by multiplying by the planned total MU, and then summed, for each beamlet, the MU that would be delivered, while that beamlet was “open,” that is, not covered by an MLC leaf. The program produced several output files that included an output columnar listing of all the beamlets showing the planned MU, delivered MU, and difference in MU (δMU). It also created two‐dimensional maps showing the same information arranged according to beamlet location. The program also kept track, for each beamlet, of the number of segments for which it was planned to be “open” and the number that actually was “open” after the overshoot was accounted for.

The program could operate on a set of files and so could conveniently analyze all the beams used for a group of patients. Two groups were studied: six prostate patients, treated with a five field protocol, and four head and neck patients, treated with a nine field protocol. The prostate set included a total of 30 fields and 2328 beamlets. The fields each averaged 155.7 MU with 36.2 irradiated segments per field. The head and neck set included a total of 47 fields and 3271 beamlets (the number of fields exceeded 4×9 because sometimes DMLC limitations required two fields to be used from one gantry angle). These fields averaged 82.0 MU with 36.1 irradiated segments per field. All these plans were developed on Corvus V3.0 using 1 cm^2^ beamlets. Each group was analyzed with the ΔfMU parameters appropriate for their treatment at this facility: 400 MU/min to the MU prescribed for treatment. Each was also analyzed with the ΔfMU parameters appropriate for the QA filming typically done here: 400 MU/min with the MU reduced by a factor of 0.25. The ΔfMU parameters for 600 MU/min were extrapolated and applied to both groups with the MU reduced by a factor of 0.15, simulating the case of a facility that might be doing its film QA with a 12‐bit digitizer. Simulations were also done for delivery rates of 240 and 80 MU/min.

The program was also used to study the overshoot effect on DMLC files that had been used for various commissioning studies. One set consisted of a $10\times 10\;{\text{cm}}^{2}$ field with a $10\times 4\;{\text{cm}}^{2}$ central area of reduced intensity. For this test, the total fluence to each beamlet was approximated by adding its open MU to its “leakage” MU: (Total MU – open MU)× transmission factor. The purpose was to better estimate the error in dose that might occur for regions that are heavily blocked during DMLC treatments.

## RESULTS

**Figure 3:**
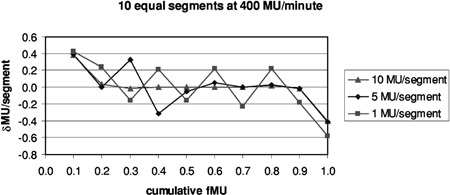
Error in delivered MU for segments receiving equal increments of MU.

Figure 3 shows the error in delivered MU/segment (δMU/segment) for three runs of the test file with equal increments of 1, 5, and 10 MU/segment. For these data, the δMU were manually computed from the fMU observed during the run. These runs were taken at 400 MU/min and show the general tendency for the first segment to receive more dose than planned, the last less, and those in between to oscillate.

**Table I:** Mean and standard error for measured ΔMU for different MU/segment ranges.

MU/minute	MU/seg<1 (MU)	1≤MU/seg<5 (MU)	MU/seg>5 (MU)
80	0.144±0.012	0.090±0.004	0.092±0.007
240	0.362±0.011	0.336±0.031	0.249±0.007
400	0.595±0.021	0.611±0.013	0.412±0.007
600	0.910	0.910	0.618

**Figure 4:**
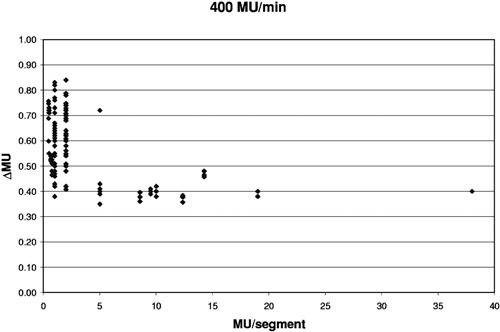
Variation of ΔMU with MU/segment when delivered at 400 MU/min.

**Figure 5:**
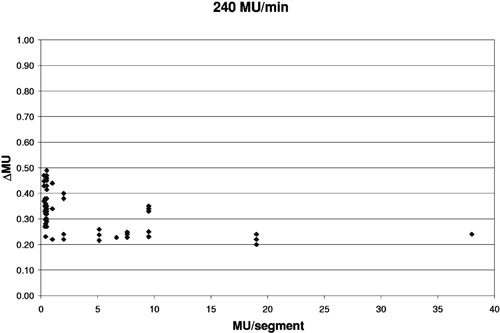
Variation of ΔMU with MU/segment when delivered at 240 MU/min.

**Figure 6:**
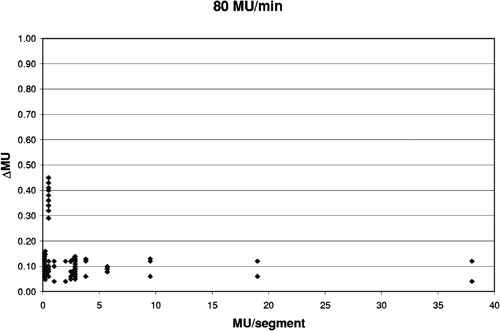
Variation of ΔMU with MU/segment when delivered at 800 MU/min.

Figures 4–6 show how the overshoot, expressed as ΔMU, varies over the range of MU/segment for the three delivery rates of 400, 240, and 80 MU/min. Each data point represents a single segment from a single run; some points overlap. The overshoot is fairly stable for >5 MU/segment and averages 0.412±0.007 MU, 0.249±0.007 MU, and 0.092±0.007 MU, respectively. These values correspond to a control loop delay of 62±1,62±2, and 69±5 msec, respectively. The overshoot becomes larger and less stable as the MU/segment decreases. For programmed MU/segment approximating the overshoot value, the test DMLC files skipped many or all segments. Thus, the data for 400 MU/min could only be obtained down to about 0.48 MU/ segment, the 240 MU/minute data down to 0.27, and 80 MU/minute data down to 0.13.

Table I shows the average ΔMU values for three ranges of MU/segment for the delivery rates measured and those extrapolated to 600 MU/min by multiplying the 400 MU/min results by 1.5. This extrapolation is justified if the delay time remains 62 msec, which is a function of the DMLC controller design.

**Figure 7:**
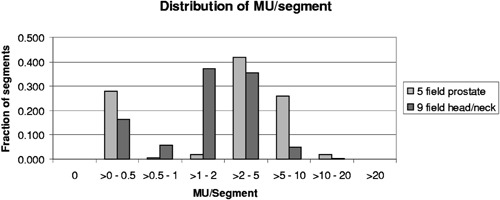
Distribution of MU/segment for plans produced by Corvus 3.0 for a 5 field prostate protocol and a 9 field head and neck protocol.

Figure 7 shows the distribution of MU/segment in the Corvus plans for the prostate and head and neck patients when treated to the full prescribed MU. 28% and 16%, respectively, of the segments, were to receive less than 0.5 MU. When the program model was applied to these fields treated at 400 MU/min, the results showed that virtually all the segments planned to receive less than 0.5 MU, would in fact be skipped.

**Figure 8:**
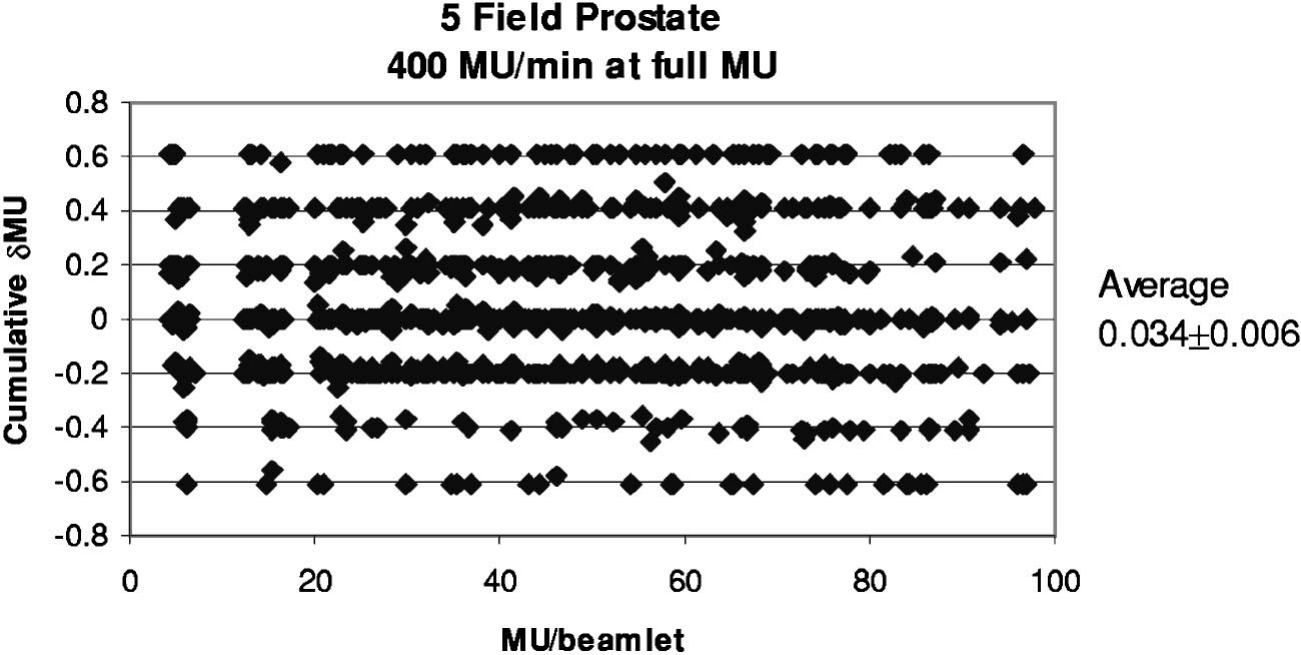
Cumulative error in delivered MU for the 2328 beamlets used in treating the prostate patients at 400 MU/min to the full prescribed MU. These values are computed using the parameters given in Table I.

**Figure 9:**
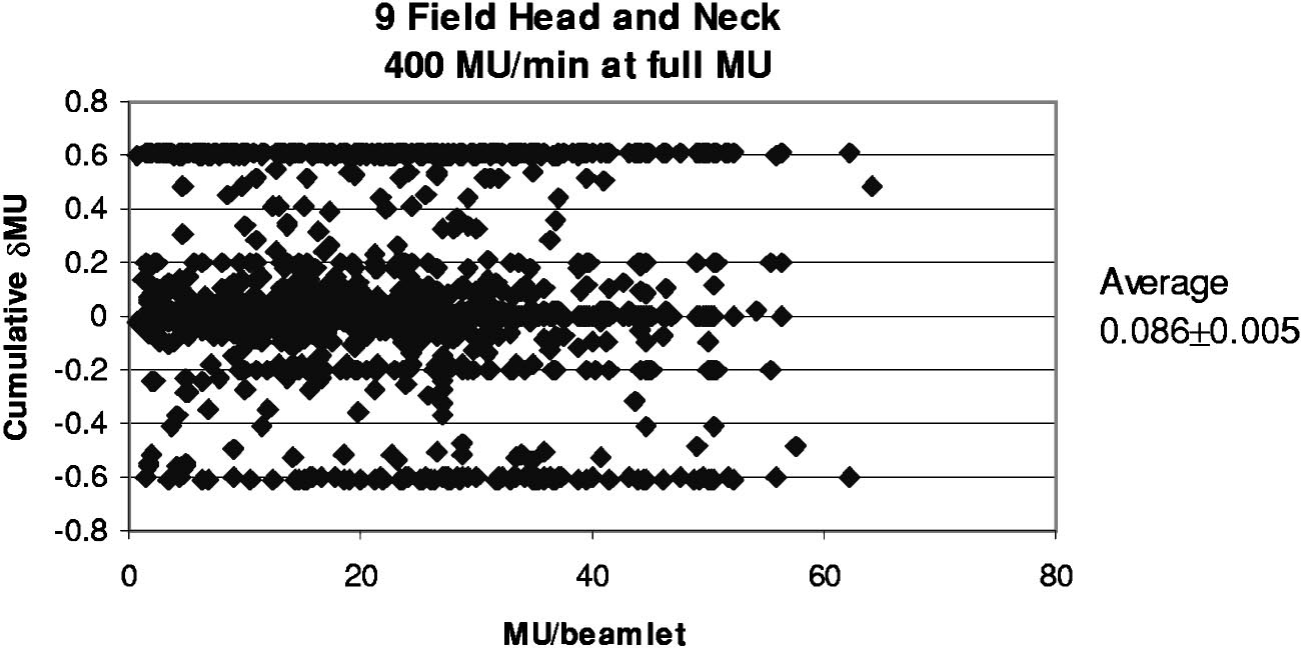
Cumulative error in delivered MU for the 3271 beamlets used in treating the head and neck patients at 400 MU/min to the full prescribed MU. These values are computed using the parameters given in Table I.

**Figure 10:**
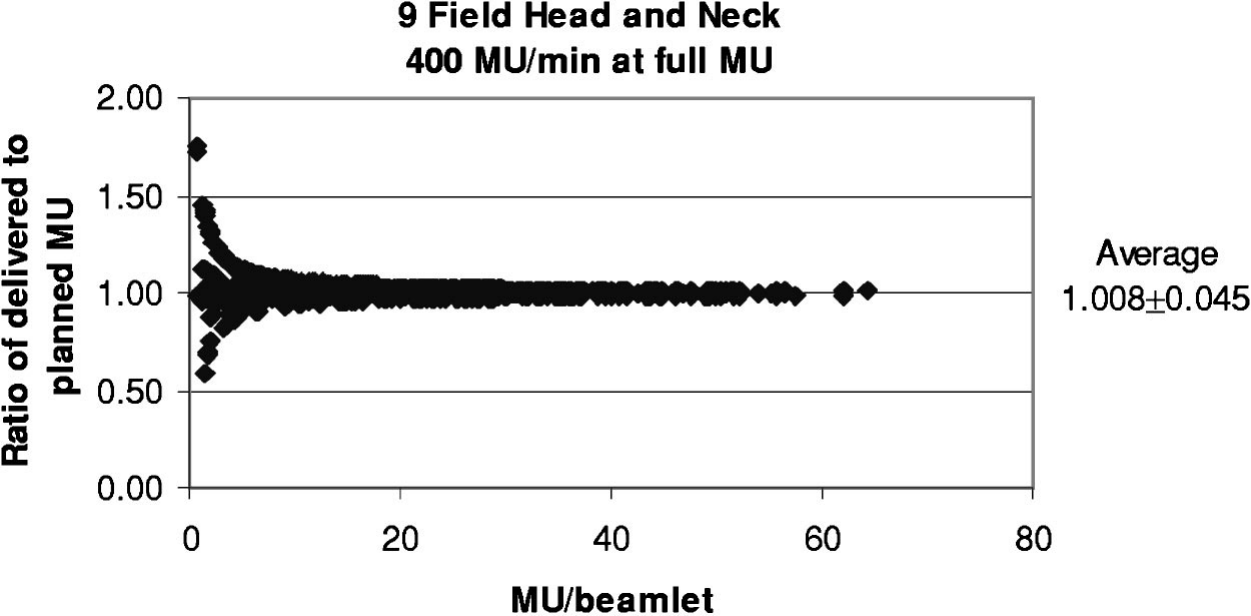
Data from Fig. 9 shown as relative error.

Figures 8 and 9 illustrate the modeled effects of the overshoot on the prostate and head and neck treatments. Each data point represents the computed error in delivered MU (δMU) to a beamlet, as a function of planned MU to that beamlet. Since several segments usually irradiate each beamlet, the cumulative δMU is the sum of the δMU of the composite segments. These may be positive or negative, as shown in Fig. 3. The average error is 0.034±0.006 and 0.086±0.005 MU, respectively. Figure 10 replots the data of Fig. 9 as the ratio of delivered to planned MU, showing that the relative error is larger for beamlets that are to receive small total MU.

**Figure 11:**
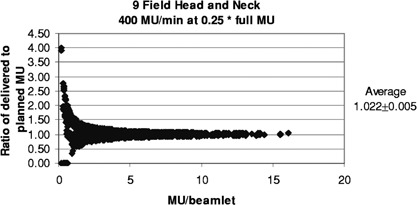
Relative error in delivered MU/beamlet to the head and neck patients with the MU reduced for filming to a dose of about 50 cGy and delivered at 400 MU/min.

**Figure 12:**
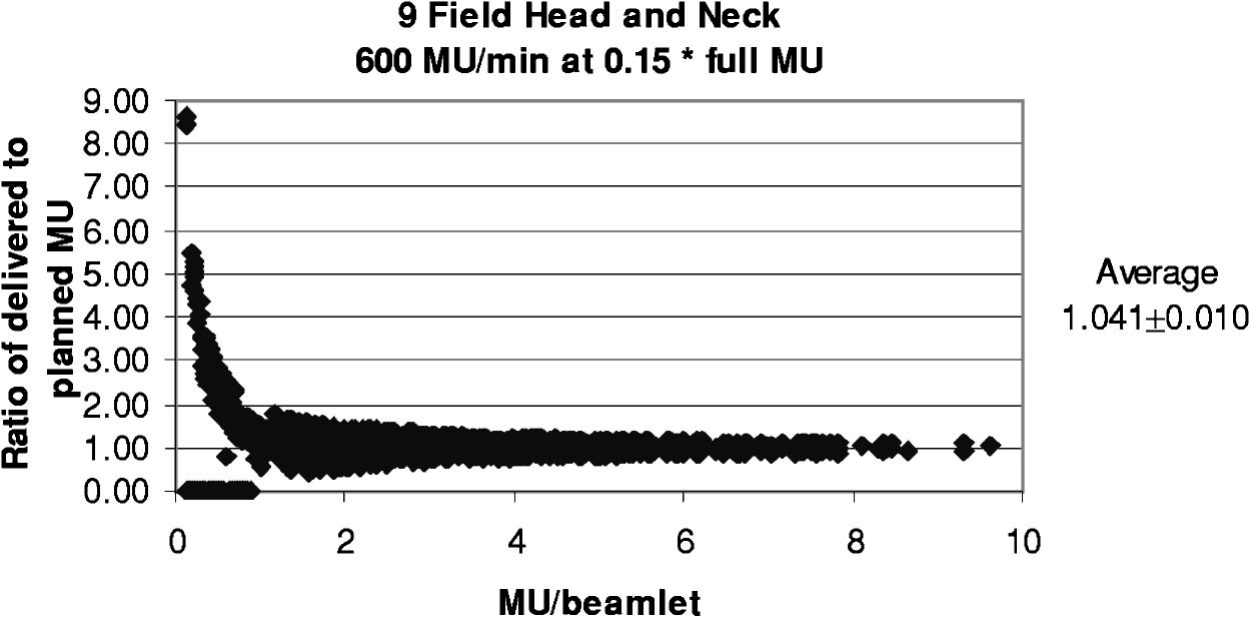
Relative error in delivered MU/beamlet to the head and neck patients with the MU reduced for filming to a dose of about 30 cGy and delivered at 600 MU/min.

**Figure 13:**
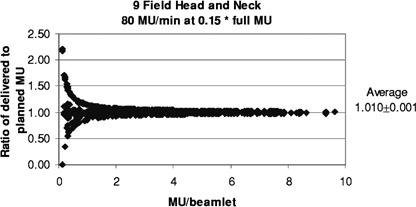
Relative error in delivered MU/beamlet to the head and neck patients with the MU reduced for filming to a dose of about 30 cGy and delivered at 80 MU/min.

Figures 11 and 12 show the results for the head and neck files when the MU have been reduced for filming. With a rate of 400 MU/min and the MU reduced by 0.25 (as done routinely here), 50% of the segments are skipped, and the ratios of delivered to planned MU/beamlet range from 0 to 4.0, with an average of 1.022±0.005. For the more extreme case of 600 MU/min and MU reduced by 0.15, 74% of the segments are skipped, and the ratios of delivered to planned MU/beamlet range from 0 to 4.0, with an average of 1.041±0.010. If a rate of 80 MU/min is used with a MU reduction of 0.15, 8.4% of the segments are skipped, and the ratios of delivered to planned MU/beamlet range from 0 to 2.2, with an average of 1.010±0.001 (see Fig. 13).

Finally, Table II shows the percent error in fluence when the commissioning test file for a 10×10 field with a 10% central “block” is irradiated at different MU/min and MU reductions.

**Table II:** Percent error in fluence for a test field: $10\times 10\;{\text{cm}}^{2}$ with 4 cm wide central area programmed to receive 10% fluence.

			Percent error in fluence	
MU/min	MU reduction factor	Left area (100%)	Central area (10%)	Right area (100%)
400	1.00	0.2	0.0	−0.2
400	0.25	1.3	4.1	−0.9
600	0.15	3.1	9.9	−2.1
80	0.15	0.3	−0.1	−0.3

## DISCUSSION

For patients treated at 400 MU/min, the data in Fig. 7 and 8 show that the average error caused by the overshoot is less than 0.1 MU. The errors are evenly distributed over beamlets receiving small to large total MU. The maximum positive error, +0.6 MU, corresponds to that associated with the first segment, and the maximum negative error, −0.6 MU, corresponds to that for the last. Although each beamlet is irradiated by several segments, the errors are not seen to accumulate, but instead tend to cancel, as is shown in Fig. 3 and suggested by clinical QA results.

This simple simulation replaced a distribution of ΔMU with averages. In Fig. 8, the banding at ±0.2 and ±0.4 MU is caused by the difference between the average values in ΔMU for segments having ≥ MU (0.4 MU) and those receiving < 5 MU (0.6 MU). For any segment, the MU error is the difference between the incoming and outgoing overshoots, which in this case happens in units of 0.2. Intermediate values occur when, for example, segments are skipped entirely. If the ΔMU values were chosen from a continuous distribution instead of three averages, then the data in Figs. 8 and 9 would be less quantized and have occasional maximum errors of about 0.85 MU, corresponding to the maxima seen in Fig. 4. One would expect the average to remain close to zero.

These data for all patients and all beamlets do not show how individual beamlets sum within a patient. Although not shown here, inspection of the program's output arrays for each beam shows that the maximum positive errors typically occur on one side of the field, corresponding to the first segment. Conversely, the maximum negative errors occur on the opposite side, corresponding to the last segment. This will be the case for leaf sequencing algorithms that systematically move the segments from left to right (or right to left) across the field. In a multi‐field plan with axial beams, these errors will tend to superimpose and cancel.

Since the errors do not tend to accumulate and are, at worst, on the order of 1 MU/beamlet, then the effect is insignificant in high dose regions. In order to make a worst case estimate of the effect on regions to be spared, consider a voxel in a critical structure irradiated with *N* beams. In the unlikely event that all the errors were positive (structure is on the leading edge of all fields), then it might receive ~NMU more than planned if one irradiates at 600 MU/min. This worst case estimate is proportional to the delivery rate.

Table II shows that some differences between ion chamber and film dosimetry in commissioning studies may be a consequence of reducing the MU for the filming, especially in low dose regions.

For all of these reasons, clinical treatments at 400 or 600 MU/min should not be compromised by this overshoot effect. There is the potential, however, for QA filming studies to be affected, as shown in Figs. 11 and 12, and Table II. Based on these results, we changed our procedures to use 240 MU/min when doing IMRT verification films using V film and an MU reduction factor of about 0.25. More recently, we have begun using Kodak EDR2 film that permits QA filming using the patient's planned MU, thus obviating many of these concerns.7 In addition, it should be noted that these results may not apply to newer designs of the DMLC control system.

## ACKNOWLEDGMENT

The authors thank Calvin Huntzinger from Varian for helpful explanations about the DMLC control system.
